# Sequential Application of Lugol’s Iodine Test after Acetic Acid for Detecting Cervical Dysplasia: A Prospective Cohort Study

**DOI:** 10.3390/diagnostics11091598

**Published:** 2021-09-02

**Authors:** Günther A. Rezniczek, Samira Ertan, Sadia Rehman, Clemens B. Tempfer

**Affiliations:** Department of Obstetrics and Gynecology, Marien Hospital Herne, Ruhr-Universität Bochum, 44625 Herne, Germany; samira.ertan@live.de (S.E.); sadia.rehman@elisabethgruppe.de (S.R.); clemens.tempfer@rub.de (C.B.T.)

**Keywords:** Lugol’s iodine test, diagnostic accuracy, colposcopy, HSIL, LSIL

## Abstract

Applying Lugol’s iodine solution to the cervix followed by colposcopic assessment is an established standard test to identify low grade/high grade squamous intraepithelial lesions (LSIL/HSIL). Here, we assessed the performance of Lugol’s iodine test during colposcopy using a standardized protocol with 5% acetic acid followed by 5% Lugol’s iodine solution and recording the most severe acetowhite (MSAWL) and iodine-negative (MSINL) lesions in a prospective cohort of consecutive women referred to our specialized colposcopy unit. The primary study endpoint was the sensitivity/specificity of MSINL for the detection of LSIL/HSIL. Secondary endpoints were the time to first appearance of the MSINL, MSINL staining intensity, and fading of MSINL. Three hundred and twenty women were included. The sensitivity and specificity of MSINL for the detection of LSIL/HSIL was 81.4 (95%—confidence interval (CI) 77.3–85.0)% and 29.5 (24.2–35.5)%, respectively. Ninety-six MSINL were identified exclusively by Lugol’s iodine test (no pathology, *n* = 46; LSIL, *n* = 29; HSIL, *n* = 21) (number needed to biopsy to identify one additional LSIL/HSIL = 1.9). In 17/320 (5.3%) patients, the clinical management was changed based on the result of Lugol’s iodine test. Video analysis showed an instant appearance of the MSINL within 10 s in 100% of cases. Intensity of MSINL significantly correlated with the presence/absence of LSIL/HSIL (Spearman rank order correlation; *p* < 0.0001). Fading of iodine-induced staining intensity over time was not observed. Thus, Lugol’s iodine showed moderate sensitivity and poor specificity, but changed clinical management in 5% of cases when used in addition to acetic acid.

## 1. Introduction

Cervical dysplasia is an important health issue affecting women of all ages [1,2]. Detecting and removing high-grade squamous intraepithelial lesions (HSIL) before they develop into invasive cervical cancer is the main goal of cervical cancer screening and an important goal of medical care of women worldwide [3,4]. Both cervical neoplasia and cervical cancer have profound impacts on the health and quality of life of affected women [5,6]. Several strategies have been developed for the prevention and early detection of cervical dysplasia, among them human papilloma virus (HPV) vaccination, HPV testing, and cervical cytology using Papanicolaou smears [1,7,8,9]. For further evaluation of women with suspected cervical dysplasia, cervical colposcopy is the standard diagnostic instrument as recommended by professional societies when at least one abnormal cytological result is observed [10,11,12]. The goal of colposcopy is to visualize cervical dysplastic lesions and to obtain a colposcopically-guided cervical biopsy for histological proof of dysplasia [10,11,13,14]. Visual inspection, the acetic acid test, and Lugol’s iodine test are the three means used for identifying cervical lesions before obtaining a cervical biopsy [10,13,15,16]. The acetic acid test has been adequately studied in prospective diagnostic trials establishing its clinical validity [17]. Surprisingly, however, there are no prospective controlled data describing the validity of Lugol’s iodine test, although colposcopists have used this test for decades as a standard test in the assessment of cervical dysplasia. In a PubMed literature search (search date 25 May 2021; search terms: colposcopy, iodine test, Lugol’s iodine test, cervical dysplasia), for example, no prospective controlled trial was identified. Therefore, we performed a prospective study with a uniform definition of each colposcopy step and an adequate number of patients and aimed to define for the first time the validity of Lugol’s iodine test in women with suspected cervical dysplasia. The primary study endpoint was the sensitivity/specificity of the most severe iodine-negative lesion (MSINL) for the detection of LSIL/HSIL. Secondary study end points included the time to first appearance of the MSINL, MSINL staining intensity, fading of MSINL, and the sensitivity/specificity of Lugol’s iodine test to identify healthy squamous epithelium. The goal of our study was to provide evidence-based data on Lugol’s iodine test, allowing colposcopists to make an informed and rational use of this test in their clinical practice.

## 2. Patients and Methods

### 2.1. Study Design, Setting, and Participants

In this prospective cohort study, we recruited consecutive women referred to the colposcopy outpatient unit of the Department of Obstetrics and Gynecology of Ruhr-Universität Bochum, Germany, between May 2020 and May 2021 for colposcopic assessment of a pathological Pap smear. After having obtained written informed consent to participate in this study, a detailed personal history was taken and documented. All colposcopists were certified specialists with a personal experience of more than 300 colposcopies. A previous excisional procedure was not an exclusion criterion. If the colposcopy was judged “adequate” by the colposcopist and if the transformation zone was adequate, i.e., either a T1-, T2-, or T3-type, then the patient was eligible irrespective of her personal history of a previous excisional procedure of the cervix. The study was approved by the Ethics Committee of the Medical Faculty of Ruhr-Universität Bochum, Bochum, Germany (registration number 20-6880).

### 2.2. Test Methods and Variables

We used a standardized colposcopy protocol with 5% acetic acid (AA) and 5% Lugol’s iodine solution in all women as follows: Using a binocular colposcope (Model 1 D LED, Leisegang, Berlin, Germany), the native impression of the cervix was noted with a 7.5× and a 15× magnification. Then, AA 5% was applied to the cervix with three pushes using a commercially available household spray can (Wilpeg, Großenlüder, Germany), corresponding to a total volume of 3 mL. Then, the colposcopist assessed and classified the most severe acetowhite lesion (MSAWL) one minute after the application of AA [17], using International Federation for Cervical Pathology and Colposcopy Rio classification criteria [18]. Then, Lugol’s iodine solution (a 5% solution of potassium iodide with iodine in distilled water) was applied to the cervix with a hand swab (Raucotupf^®^, Lohmann & Rauscher, Lengsdorf, Germany) and all iodine-negative lesions (INL) were recorded. Biopsies were taken from the MSAWL and additional INL (if present). Endocervical samples using a small Kevorkian’s curette were obtained in case of a transformation zone type 3 and/or a MSAWL/INL protruding into the endocervix. All colposcopies were video-documented.

### 2.3. Analysis

Based on the video material, two colposcopists retrospectively and independently graded the staining intensity of the most severe INL (MSINL) using a 3-tier grading system (intense, intermediate, and minimal yellow staining). In addition, the time between the application of Lugol’s iodine solution and the first appearance of the MSINL and the time from application of Lugol’s iodine solution to the start of fading of iodine-negative yellow staining (i.e., the continuous loss of iodine staining intensity over time) of the MSINL were recorded. The primary study endpoint was the sensitivity/specificity of the MSINL for the detection of LSIL/HSIL, based on the histopathology reports of the individual samples. Secondary study endpoints were as follows: the number of additional LSIL/HSIL detected by Lugol’s iodine test, the number of biopsies needed to detect one additional LSIL/HSIL, and, based on the analysis of colposcopy videos, the time to first appearance of the MSINL, the staining intensity of the MSINL and its correlation with the presence of LSIL/HSIL, fading of MSINL, and the sensitivity/specificity of a positive iodine test (i.e., dark brown staining) to identify healthy squamous epithelium.

### 2.4. Study Size

The sample size calculation assumed that the diagnostic validity of Lugol’s iodine test would be rejected if an additional LSIL/HSIL was detected in <1% of a given patient population when Lugol’s iodine test was used in addition to AA. Using the certification criteria of the German Cancer Society (DKG) for the certification of a so-called dysplasia unit, a yearly volume of at least 300 colposcopies is required [19,20]. Based on this number regarded as representative of the yearly volume of a specialized dysplasia outpatient clinic, we aimed to recruit 320 patients assuming a 5% rate of patients with protocol violations, screening failures, or lost to follow-up.

### 2.5. Data Management and Statistical Methods

Study data were collected and managed using REDCap (Research Electronic Data Capture), a secure, web-based application designed to support data capture for research studies [21]. After data collection had been concluded, exported data were further processed in Microsoft Excel (Microsoft Inc., Redmond, WA, USA) and prepared for statistical analyses using SigmaPlot 14.5 (Systat Software Inc., San Jose, CA, USA). Descriptive statistics were reported using means and standard deviations for normally distributed data and medians and interquartile ranges (IQR) for data not meeting this assumption. Accordingly, statistical analysis was performed using parametric (*t*-test) or nonparametric (Mann–Whitney U-test) tests. To compare rates and proportions, the χ^2^-test was used. All *p*-values were two-tailed and *p* < 0.05 was considered statistically significant.

## 3. Results

### 3.1. Patients, Colposcopies, and Biopsies

**Figure 1:**
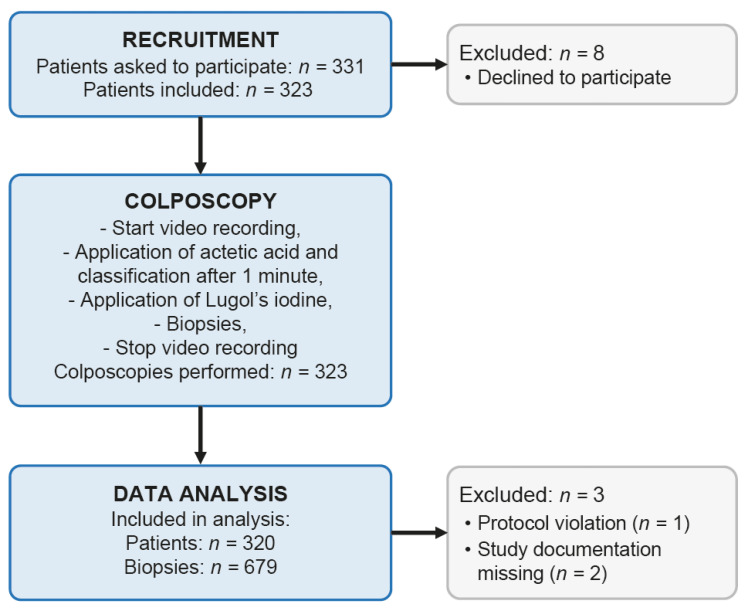
Flow diagram of the study and analysis.

We prospectively recruited 323 consecutive patients. Of these, one case was excluded due to a protocol violation (no samples collected). In two cases, the study documentation was missing. Thus, data from 320 patients were included in the study. Figure 1 gives a detailed account of the patients’ flow through the study and the workflow. Patient characteristics, Pap smear results, the colposcopic findings after application of AA and Lugol’s iodine solution, respectively, and the corresponding histopathological results are shown in Table 1. All patients underwent a standardized colposcopy protocol with AA and Lugol’s iodine solution as delineated in Section 2. At the end of colposcopy, i.e., after both AA and Lugol’s iodine, colposcopic assessment found a normal cervix in 84 (26.3%), minor changes in 137 (42.8%), major changes in 97 (30.3%), major changes suspicious for invasion in 1 (0.3%), and non-specific changes in 1 (0.3%) of the cases, respectively. Type 1, type 2, and type 3 transformation zones were identified in 182 (57.6%), 43 (13.6%), and 91 (28.8%) cases, respectively. A total of 679 cervical biopsies were collected, ranging from 0 to 4 per patient with a median of 2 (interquartile range 1–3); details are given in Table 2. Of note, we observed no case of allergic reaction to iodine in any of the included patients.

### 3.2. Sensitivity and Specificity of Lugol’s Iodine to Detect LSIL/HSIL

We were interested in defining the sensitivity and specificity of Lugol’s iodine test for the detection of LSIL/HSIL. This was the primary endpoint of the study. We found that the sensitivity of Lugol’s iodine test was 81.4 (95%—confidence interval (CI) 77.3–85.0)% and the specificity was 29.5 (24.1–35.5)%. Specifically, Lugol’s iodine test identified 96 lesions, 50 of which were LSIL or HSIL (29 LSIL, 21 HSIL) in addition to AA (Table 3). To identify these additional LSILs/HSILs, 96 biopsies in 66 patients were taken based on iodine-negative lesions not already identified by AA. Therefore, the mean number needed to biopsy to obtain one additional LSIL/HSIL was 1.92. A clinical benefit (defined as management change) based on the result of Lugol’s iodine test, however, was only seen in 17 of the 66 patients, because in the other 49 patients, AA had already identified an LSIL/HSIL either in the same cervical quadrant (*n* = 14) or in another cervical quadrant (*n* = 19), or LSIL/HSIL was detected in the ECC sample (*n* = 16). Thus, a management change based exclusively on Lugol’s iodine test was observed in 5.3% (17/320; 8 HSIL, 9 LSIL) of patients with 5.6 additional biopsies necessary for 1 altered management decision.

### 3.3. Optimal Time Point for the Assessment of Iodine Staining

**Figure 2:**
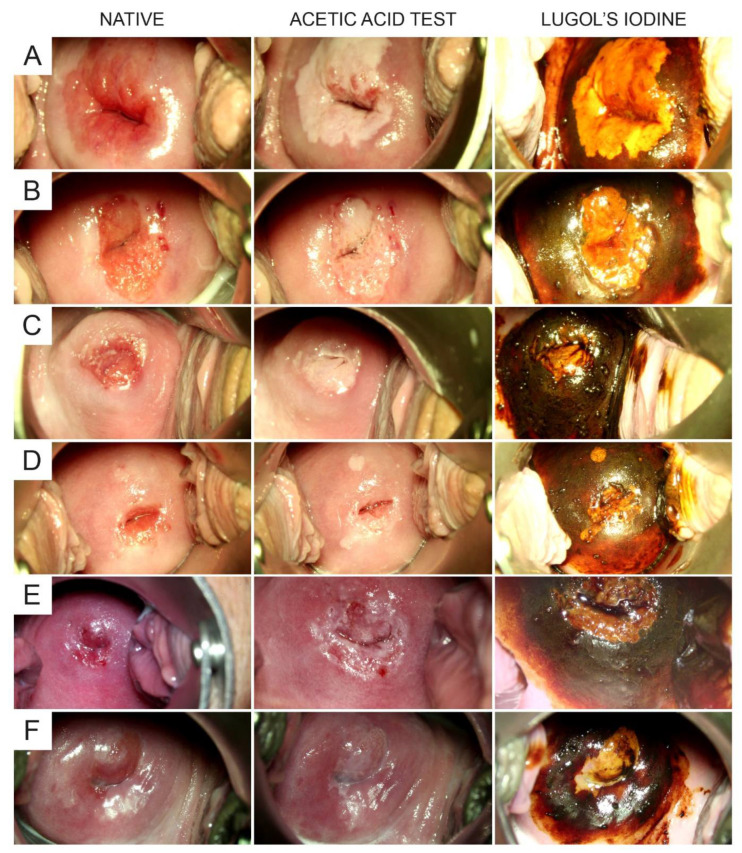
Video stills showing in each row the native cervix (left column), the cervix approx. 1 min after the application of acetic acid (middle column), and the cervix within 10 s after the application of Lugol’s iodine (right column), respectively. Each row (**A**–**F**) represents a different patient.

We were also interested in defining the optimal time point for the colposcopic assessment of a MSINL after the application of Lugol’s iodine solution. Thus, we analyzed the time between the application of Lugol’s iodine solution and the appearance of the MSINL. Video analysis in a subset of recordings (*n* = 233) showed an instant appearance of the MSINL within 10 s or an instant dark brown staining (in case no iodine-negative lesion was present; *n* = 73) in 100% of patients (Table 4). Therefore, the optimal time point for the colposcopic assessment of the iodine test based on our study was 10 s after the application of Lugol’s iodine solution. Furthermore, we evaluated the validity of grading the staining intensity of the MSINL. When we looked at the video assessments of two colposcopists, there was a fair congruence regarding their judgements of MSINL staining intensity (Table 4). We found that grading the intensity of MSINL (no yellow staining versus minimal, intermediate, or intensive yellow staining) was highly significantly correlated with the presence of histopathology (no dysplasia versus LSIL, HSIL, or invasive carcinoma) with Spearman rank order correlation coefficients of 0.300 (rater 1) and 0.308 (rater 2), respectively (both: *p* < 0.0001). However, for neither rater did the intensity of the yellow staining clearly differentiate between LSIL and HSIL (*p* > 0.05; Table 4). Lastly, we were interested in ruling in or ruling out the potential phenomenon of “fading”, i.e., the continuous loss of iodine staining intensity over time, as previously described for other tests used in colposcopy such as the AA test. We found that fading was not present in any of the cases with iodine negativity (yellow staining) included in the detailed video analysis (Table 4). Figure 2 shows representative series of stills taken from the colposcopy videos, each showing the native cervix and the cervix ~1 min after the application of AA and within 10 s after the application of Lugol’s iodine solution, respectively.

## 4. Discussion

Lugol’s iodine test of the cervix during colposcopic assessment of women with suspected cervical dysplasia has been used for decades as a standard test in clinical practice [22]. However, there are no prospective controlled data describing the validity of this test. Therefore, we performed a prospective cohort study and found that Lugol’s iodine test had a sensitivity and specificity for detecting LSIL/HSIL of 81.4% and 29.5%, respectively, and led to a clinical management change in 5% of patients when used in addition to AA. A mean of 1.9 additional cervical biopsies had to be performed for detecting one additional LSIL/HSIL. Of note, the intensity of iodine staining highly significantly correlated with histopathology suggesting biological plausibility of this test.

The use of AA for the colposcopic detection of cervical dysplasia is well established and has a high sensitivity and specificity [17,23,24]. Therefore, it is questionable that an additional test such as Lugol’s iodine makes sense and adds to the diagnostic yield of colposcopy and AA. We can now say that based on the results of this study, Lugol’s iodine test does significantly add to the diagnostic performance since it identified additional LSIL/HSIL not seen by AA in 66/320 women. The mean number needed to biopsy to obtain one additional LSIL/HSIL was only 1.92. In every twentieth woman, this led to a change in clinical management. Therefore, Lugol’s iodine test should be a part of the routine diagnostic algorithm of colposcopy in all women with suspected cervical dysplasia.

Our study also defines, for the first time, specific features of Lugol’s iodine test such as the fact that the effect of staining with iodine is apparent in all patients within 10 s and that there is no fading, a phenomenon well known for the AA test. The use of Lugol’s iodine test is not only diagnostically useful, but also biologically plausible. This is underlined by our finding that grading of the intensity of iodine-stained areas highly significantly correlated with the results of histopathology (no dysplasia versus LSIL, HSIL, or invasive carcinoma; *p* < 0.0001).

In summary, the results of our study add to the literature describing an evidence-based and rational use of diagnostic tests for the work-up of women with cervical dysplasia. This is of clinical relevance because cervical dysplasia is a common disorder affecting millions of women worldwide [1,2,3,4,7]. We propose that Lugol’s iodine test should be performed subsequent to the AA test in all women undergoing colposcopy for suspected cervical dysplasia. In every twentieth woman, this test results in a clinical benefit consisting of a management change based on the test results. However, it has to be kept in mind that the assessment of any colposcopic finding, performed by expert colposcopists with specific and reproducible methodology, must always take into account the patients’ features and the reference cytology in addition to the test results of the AA test and Lugol’s iodine test.

Our study has strengths and limitations. The study strengths include the high number of patients, the prospective design, and a standardized colposcopy algorithm, which was defined as part of the study protocol. Moreover, we used video documentation of all procedures confirming a uniform use of all diagnostic measures and all procedures were performed by experienced colposcopists, thus excluding bias due to learning curve effects [25,26]. Our study also has limitations, among them possible selection bias. Our study population may not be representative of other patient populations seen by gynecologic clinics and practices because we only included women referred to our specialized colposcopy unit by a group of gynecologic practices pre-selecting patients for specialized care. Therefore, the likelihood of finding LSIL/HSIL by Lugol’s iodine test may be higher compared to other settings. Specifically, our population may not be representative of other populations in other countries regarding important characteristics such as age, reference cytology, HPV status, and possible history of immunosuppression such as HIV. Such differences, if present and if quantitatively relevant, may influence the validity of Lugol’s iodine test. In addition, self-selection bias is possible due to patients insisting on being referred to a specialized colposcopy unit.

Lugol’s iodine test is a commonly used diagnostic tool when colposcopically assessing the cervix in order to identify cervical dysplasia [10,13,22]. However, there is a lack of data exactly defining how valid this test is when used in addition to the AA test, which is the standard test used in every colposcopy [10,11,12,17]. Some colposcopists use Lugol’s iodine test routinely, some only in selected cases, and others only when assessing the vaginal wall for potential vaginal intraepithelial lesions. In any case, these practice patterns are based on personal experience. Our data show that Lugol’s iodine test is a reasonable addition to the AA test since it adds valuable information in a considerable number pf patients and leads to a change of management decisions in 5% of them. Therefore, we interpret our data as the basis for a recommendation to use Lugol’s iodine test sequentially after the AA test in all women undergoing colposcopic assessment of the cervix. It is of note that within the context of this study, we did not routinely perform endocervicoscopy, a technique used to hysteroscopically visualize the endocervix in women with abnormal cervical cytology and a type 3 transformation zone. This technique uses endoscopic examination of the endocervical epithelium with office-based continuous-flow hysteroscopy after application of acetic acid 5%, followed by targeted biopsies [27,28,29]. This has to be kept in mind when extrapolating the results of our study to local practice practicing routine endocervicoscopy.

## 5. Conclusions

In conclusion, Lugol’s iodine test during colposcopy showed moderate sensitivity and poor specificity, but changed clinical management in 5% of cases when used in addition to acetic acid. Intensity of iodine staining significantly correlates with the presence/absence of LSIL/HSIL. Therefore, Lugol’s iodine test should be a part of the standard diagnostic algorithm of colposcopy in all women with suspected cervical dysplasia.

## Figures and Tables

**Table 1:** Patient characteristics, colposcopy, and histopathology.

Patient Characteristic	Value
Number of patients	320
Age (years)	37.6 (32.1–45.5), range 20.4–83.6
Body mass index (kg/m^2^)	23.8 (21.4–28.0), range 16.0–48.6 (*m* * = 4)
Parity	1 (0–2), range 0–6 (*m* = 3)
Allergies (yes/no)	102 (32.0%)/217 (68.0%) (*m* = 1)
Currently smoking (yes/no)	145 (45.9%)/171 (54.1%) (*m* = 4)
Ever smoked (yes/no)	178 (56.3%)/138 (43.7%) (*m* = 4)
Alcohol abuse (yes/no)	6 (1.9%)/312 (98.1%) (*m* = 2)
Drug abuse (yes/no)	13 (4.1%)/306 (95.9%) (*m* = 1)
Concomitant disease (yes/no)	131 (41.1%)/188 (58.9%) (*m* = 1)
Number of concomitant diseases	0 (0–1), range 0–4 (*m* = 1)
Prescription drug use (yes/no)	177 (55.5%)/142 (44.5%) (*m* = 1)
Number of prescription drugs	1 (0–1), range 0–12 (*m* = 1)
Immunosuppressive conditions (yes/no)	18 (5.7%)/299 (94.3%) (*m* = 3)
Type of transformation zone	
Types 1/2/3	182 (57.6%)/43 (13.6%)/91(28.8%) (*m* = 4)
Indication for colposcopy	
ASC-H	21 (6.6%)
AGC endocervical favor neoplastic	5 (1.6%)
LSIL	121 (37.8%)
HSIL	173 (54.1%)
HPV status (pos/neg/unknown)	197 (61.5%)/20 (6.3%)/103 (32.2%)
Colposcopic findings (after acetic acid test; RIO classification)	
Normal	84 (26.3%)
Minor changes	137 (42.8%)
Major changes	97 (30.3%)
Suspicious for invasion	1 (0.3%)
Non-specific	1 (0.3%)
Colposcopic findings (after Lugol’s iodine)	
Iodine positive (dark brown staining only)	73 (22.8%)
Iodine negative (yellow staining **)	247 (77.2%)
Histological results	
Negative for dysplasia	77 (24.1%)
LSIL	88 (27.5%)
HSIL	151 (47.2%)
AIS	3 (0.9%)
Invasive cancer	1 (0.3%)

Values are counts or medians (interquartile ranges). AIS, adenocarcinoma in situ; AGC, atypical glandular cells; AGC-NOS, AGC not otherwise specified; ASC, atypical squamous cells; ASC-H, ASC cannot exclude HSIL; ASC-US, ASC of undetermined significance; HPV, human papilloma virus; HSIL/LSIL, high/low grade squamous intraepithelial lesions; n/a, not applicable, Pap, Pap smear. * Number of missing values. ** Yellow staining in at least one quadrant of the cervix.

**Table 2:** Cervical biopsies.

Item	Value
Number of patients	320
With 0 cervical biopsies (ECC only)	10 (3.1%)
With 1 cervical biopsy	76 (23.8%)
With 2 cervical biopsies	107 (33.4%)
With 3 cervical biopsies	119 (37.2%)
With 4 cervical biopsies	8 (2.5%)
Cervical biopsies per patient	2 (1–3); range 0–4
Number of cervical biopsies	679
Acetic acid test positive	469 (69.1%)
Lugol’s iodine test positive *	524 (77.2%)
Both tests positive	428 (63.0%)
Both tests negative	114 (16.8%)
With pathologic finding	415 (61.1%)
Acetic acid test positive	316 (76.1%)
Lugol’s iodine test positive*	338 (81.4%)
Both tests positive	288 (69.4%)
Both tests negative	49 (11.8%)

Values are counts or medians (interquartile ranges). ECC, endocervical curettage. * Defined as iodine-negative, yellow staining.

**Table 3:** Primary and secondary study outcomes.

Outcome Item	Value
Sensitivity and specificity of Lugol’s iodine to identify LSIL/HSIL	
Sensitivity, %	81.4 (77.3–85.0)
Specificity, %	29.5 (24.2–35.5)
PPV, %	64.5 (60.2–68.6)
NPV, %	50.3 (42.2–58.4)
Accuracy, %	61.3
Additional lesions identified by Lugol’s iodine test *	96
No pathology	46 (47.9%)
LSIL	29 (30.2%)
HSIL **	21 (21.9%)
Number needed to biopsy	1.92
Number of patients with at least one additionally identified lesion by Lugol’s iodine test*	66/320 (20.6%)
Clinical management change based on Lugol’s iodine ***	17/320 (5.3%)
Sensitivity and specificity of Lugol’s iodine to identify healthy epithelium	
Sensitivity, %	29.5 (24.2–35.5)
Specificity, %	81.4 (77.3–85.0)

Values are counts or percentages (95% confidence intervals). * In addition to those identified by the acetic acid test. ** Of these, 11 (52.4%) were positive for high-risk HPV, and in 10 (47.6%), HPV status was unknown. *** Pathologic finding in a biopsy identified by Lugol’s iodine when no lesions identified by the acetic acid test or endocervical curettage (in case of a type 3 transformation zone) with pathologic findings were present in a patient. HSIL/LSIL, high/low grade squamous intraepithelial lesions; PPV/NPV, positive/negative predictive value.

**Table 4:** Video analysis.

Item	Value	
Number of videos analysed, *n*	233	
Grading of Lugol’s iodine test	Rater 1	Rater 2
No iodine-negativity (dark brown staining only), *n* (%)	42 (18.0%)	32 (13.7%)
Iodine negative (yellow staining), * *n* (%)	191 (82.0%)	201 (86.3%)
Minimal yellow staining (grade 1)	50 (26.2%)	14 (7.0%)
Intermediate yellow staining (grade 2)	40 (20.9%)	52 (25.9%)
Intense yellow staining (grade 3)	101 (52.9%)	135 (67.2%)
Inter-rater agreement **		
Exact, %	60.1	
Grades 0/1 vs. grades 2/3, %	76.8	
Max. difference of 1 grade, %	88.4	
Fleiss’s kappa	0.47 (95%-CI: 0.38–0.55) [moderate]	
Cronbach’s alpha	0.83 [good]	
Correlation of grading and lesion severity	Rater 1	Rater 2
Spearman rank order correlation, *** CC; P	0.300; <0.00001	0.308; <0.00001
Chi square test ****, P	0.295	0.078
Fading of MSINL (yes/no), *n* (%)	0 (0%)/191	0 (0%)/201
Time to first appearance of MSINL within 10 seconds (yes/no), *n* (%)	191 (100%)/0	201 (100%)/0

* Most significant iodine-negative lesions. ** Expressed as percentage proportion of agreement, either exact agreement or with a maximal difference of 1 grade level (no iodine-negativity = grade 0), or as Fleiss’s free-marginal multi-rater kappa or Cronbach’s alpha. *** Grading of Lugol’s iodine test versus histopathology (0 = no pathology, 1 = LSIL, 2 = HSIL, 3 = cancer). **** χ^2^ test: staining intensity (minimal/intermediate and intense) versus histopathology (LSIL, HSIL), excluding false positives and true negatives. CC, correlation coefficient; CI, confidence interval; MSINL, most severe iodine-negative lesion; HSIL/LSIL, high-/low-grade squamous intraepithelial lesions.

## Data Availability

Data will be shared upon reasonable request made to the corresponding author. This includes individual participant data underlying the results presented here, after de-identification, as well as data dictionaries and the study protocol. Data is available after publication, without a specific end date. Requesting investigators must show that their proposed use of the data has been approved by an independent review committee identified for this purpose.
